# Seasonal variation of endogenous adrenocorticotropic hormone concentrations in healthy non-geriatric donkeys in Northern California

**DOI:** 10.3389/fvets.2022.981920

**Published:** 2022-08-12

**Authors:** Sarah Humphreys, Philip H. Kass, K. Gary Magdesian, Erin Goodrich, Emily Berryhill

**Affiliations:** ^1^School of Veterinary Medicine, University of California, Davis, Davis, CA, United States; ^2^Department of Population Health and Reproduction, University of California, Davis, Davis, CA, United States; ^3^Department of Medicine and Epidemiology, University of California, Davis, Davis, CA, United States; ^4^Department of Population Medicine and Diagnostic Sciences, Cornell University, Ithaca, NY, United States

**Keywords:** circannual, clinicopathology, endocrinopathy, seasonality, screening, testing

## Abstract

Elevated plasma adrenocorticotropic hormone (ACTH) is often used to diagnose pituitary pars intermedia dysfunction (PPID) in horses. The hormone naturally increases in the fall in horses, and donkeys have been found to have higher ACTH concentrations than horses. However, circannual variation of ACTH has not been assessed in donkeys. The objective of the study was to establish seasonal variation of basal plasma ACTH concentrations over the course of a year in clinically healthy, non-geriatric donkeys. It was hypothesized that donkey ACTH concentrations would be higher than those reported in horses without PPID in all seasons, and that, similarly to horses, ACTH concentrations would further increase in the fall months. Twenty-six healthy adult donkeys (10 standards, 16 miniatures), a median (range) of 6 (2–13) years of age, were included. Donkeys were housed at a single location. Serial plasma samples were obtained monthly for 12 months. Plasma ACTH concentrations were determined by immunoassay. Data are presented as median (range), with a *P*-value < 0.05 considered significant. ACTH concentrations were lowest in the winter and spring [12.8 (5.0–73.6) pg/ml and 12.5 (2.8–62.6) pg/ml, respectively], with an increase in the summer [53.2 (29.7–305.0) pg/ml], and peak in the fall [77.1 (12.4–319.0) pg/ml]. ACTH concentrations were highest in the month of September [122.0 (41.7–319.0) pg/ml]. Donkey ACTH concentrations were higher than equine reference ranges from May through November but showed similar circannual variation with dramatic increases in the fall months. Species-specific reference ranges are necessary for accurate interpretation of endocrinopathy screenings in donkeys.

## Introduction

Pituitary pars intermedia dysfunction (PPID) is the most common endocrinopathy of aging equids (1–3). Pathophysiology of this disease involves loss of hypothalamic dopaminergic inhibition of the pars intermedia (1). This results in increased production of pars intermedia hormones derived from pro-opiomelanocortin, including adrenocorticotropic hormone (ACTH) (3). The disease is diagnosed from clinical signs, assessment of basal plasma ACTH concentrations alone, and/or in conjunction with a thyrotropin releasing hormone stimulation test (4, 5). Diagnosis is complicated by seasonal variation of plasma ACTH concentrations, with those rising in the fall months in both affected and unaffected horses (6). Reference ranges for seasonal variation in horses have been established in select locations, however geographic location (latitude and longitude) is known to influence values as well (7).

The majority of the research on PPID in Equidae is focused on horses, with limited data on the endocrinopathy in donkeys (8). While donkeys have been steadily gaining in popularity as companion animals in affluent countries, decisions regarding their care are generally extrapolated from the body of knowledge of horses (9). However, there are many differences between the two species ranging from drug metabolism to nutritional requirements, with additional disparities in hematologic and clinical pathologic reference ranges (8, 10–17). Diagnosis of PPID in donkeys through clinical signs such as hypertrichosis, lethargy, or a pot-bellied appearance can be more difficult than in horses because of their inherent differences in appearance and behavior (18, 19). Onset of laminitis may be the first clue of an underlying endocrinopathy in donkeys (20). A recent retrospective study of post-mortem findings in 1,444 aged donkeys revealed a prevalence of only 1.8% for PPID in this particular population, but nearly all of those also had a concurrent foot disorder such as laminitis (21).

There are few published studies evaluating donkey ACTH concentrations across seasons and with respect to geographic location. One study demonstrated that donkey ACTH concentrations are higher than those found in horses in the spring and summer but did not assess ACTH concentrations serially or in fall months (8). An additional study in Germany compared donkey to mule ACTH concentrations and found that concentrations in donkeys were higher than in mules in February, May, August, and November, with highest concentrations (62.93 ± 37.40 and 34.38 ± 16.21 pg/ml) in August, respectively (22).

The objective of the current study was to establish the seasonal variation of basal plasma ACTH concentrations over the course of a year in clinically healthy, non-geriatric donkeys. It was hypothesized that: (1) donkeys would have higher basal plasma ACTH concentrations compared to those reported in horses, and (2) the trend of seasonal variability would be similar to horses, with an increase in ACTH concentrations in the fall months.

## Materials and methods

### Animals

A total of 26 donkeys (16 miniature and 10 standard donkeys) were recruited from a private foundation, with a median (range) age of 6 (2–13) years at the time of study enrollment (March 2019). There were a total of 18 intact non-pregnant females and 8 castrated males. Of the miniature donkeys, 12 were female and 4 were castrated males, with a median (range) age of 5.5 (2–12) years. There were 6 female and 4 castrated male donkeys in the standard donkey group with a median (range) age of 4 (3–13) years. Donkeys were deemed clinically healthy based on history, physical examination, and baseline complete blood counts (CBCs) prior to enrollment. Donkeys were housed in a dry lot in northern California (relative latitude 38.534, longitude−121.824) with intermittent access to pasture and were free fed a grass hay mix with no additional supplementation. They received annual vaccinations and deworming according to their regular veterinarian recommendations. Management between both standard and miniature groups were similar.

This prospective observational study was approved by the Institutional Animal Care and Use Committee of the University of California Davis (IACUC #20894, approved December 28, 2018). Written consent for venipuncture of the donkeys was obtained from the foundation.

### Hematologic collection and analysis

Blood was drawn from the jugular vein using a 19 g or 20 g 1 1/2" BD Vacutainer needle (Exel International Medical Products) and collected into plastic EDTA tubes monthly from March 2019 through February 2020. To better assess the trends in ACTH concentrations in the summer and fall months, samples were obtained twice monthly from June through November, with a range of 13–19 days between time points. For months in which collection was performed once (December through May), venipuncture was performed between the second and third week of each month. Sampling was consistently performed in the morning (between the hours of 0800–1100 h) after a hay meal. Blood was immediately chilled and centrifuged within 2 h of collection (Thermo Scientific centrifuge, 1,684 g, 7 mins, 20°C).

Plasma was transferred to cryovials and stored at −80°C prior to shipment on ice to the Endocrinology Laboratory at the Cornell University Animal Health Diagnostic Center (AHDC). March through May samples were analyzed using the Immulite 1000 immunoassay (Siemens Medical Solutions USA, Inc.), previously validated for use in equids by the laboratory [reference range for horses, 9–35 pg/ml (https://app.vet.cornell.edu/ahdc-portal/test-fee/details?test_code=ACTHIN)] and a limit of detection of 2 pg/ml. The remainder of the samples (June through January) were analyzed using the Immulite 2000 immunoassay (reference range, 2–30 pg/ml). The values reported from March through May were converted to the comparable Immulite 2000 values using the following equation provided by the AHDC and generated for use in horses: Imm2000 = (0.998 × Imm1000) – 6.775; R^2^ = 0.959. Validation for the Immulite 2000 was performed previously at the reference laboratory using a total of 406 equine samples with a maximum ACTH concentration of ~400 pg/ml in both the Immulite 1000 and the Immulite 2000.

Blood was submitted for a CBC at the time of venipuncture in March and again in September to assess for clinicopathological signs of systemic inflammation as an additional indicator of systemic health. The CBCs were performed using an Advia 120 (Siemens Medical Solutions USA, Inc.) and manual differential count. References for CBC parameters in donkeys were derived from Burden et al. (17).

### Statistical analysis

All analyses were performed using Stata IC/15.1 (StatCorp, College Station, TX) or GraphPad Prism v. 9.3.1 (GraphPad Software). *P*-values < 0.05 were considered statistically significant, and median (range) are reported for data not normally distributed.

Mixed effects analysis of variance was used to evaluate the fixed main and interaction effects of age, month, and sex on log-transformed ACTH concentrations given the longitudinal (repeated measures) nature of the data. The individual donkey was treated as a random effect in each model. January was considered the reference month against which all other months were compared in the ANOVA models, and no *post-hoc* inter-month comparisons were performed. A benefit of mixed effects models is the allowance of missing data points throughout the sampling period. One data point in two individual donkeys was excluded from analysis due to supraphysiologic concentrations and presumptive assay error (669 and 1,250 pg/ml). Additionally, one of the miniature donkeys that was averse to venipuncture and at risk of having spuriously elevated ACTH due to stress was not sampled on one of the collection dates.

For months in which two ACTH samples were obtained, the Shapiro-Wilk test was performed to determine if the distribution of the data was consistent with normality. Data were not found to be normally distributed, and Wilcoxon matched-pairs signed rank tests were performed to evaluate for differences between concentrations obtained mid-month compared to those obtained at the end of the month.

### Seasonality

For the purposes of this study, seasonality was defined as follows: spring was classified as March through May, summer was June through August, fall was September through November, and winter was December and January. Blood was collected in February, but samples were unable to be processed due to unforeseen circumstances, leaving a total of 11 months of sampling.

## Results

### ACTH concentrations by month and season

**Figure 1:**
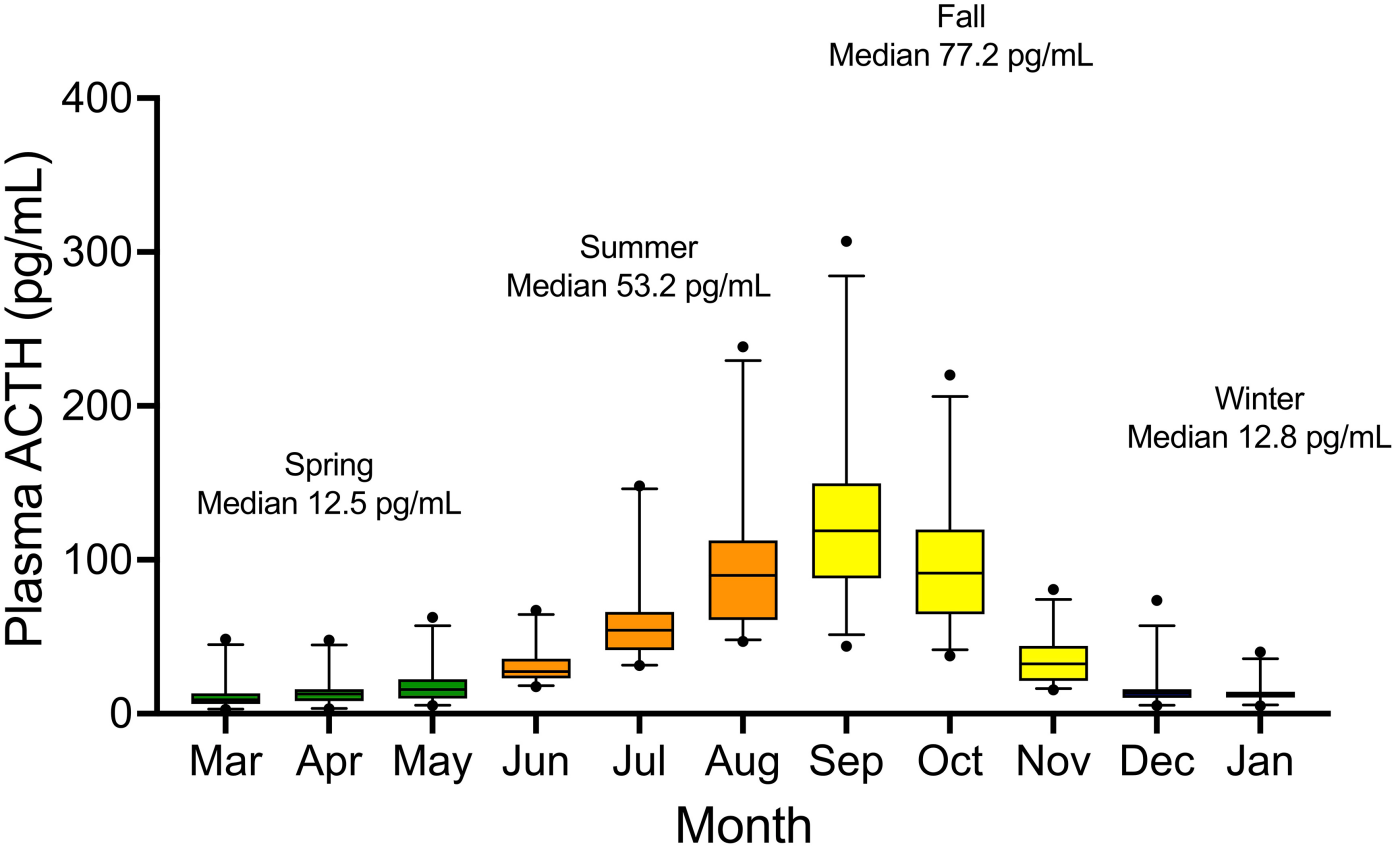
Box and whisker plot of plasma ACTH concentrations by month in 26 clinically healthy adult donkeys. 5-95th percentiles notated by bars, with outliers notated by solid circles.

Median plasma ACTH concentrations are displayed by month and grouped into seasons (Figure 1). Median (range) and 95% confidence intervals for plasma ACTH concentrations by season were as follows: spring 12.5 (2.8–62.6) pg/ml (95% CI 10.1–14.1 pg/ml), summer 53.2 (29.7–305.0) pg/ml (95% CI 46.0–58.6 pg/ml), fall 77.1 (12.4–319.0) pg/ml (95% CI 63.6–85.7 pg/ml), and winter 12.6 (9.7–15.5) pg/ml (95% CI 11.2–14.1 pg/ml). Following log10 transformation of data, month was found to be a significant variable (*P* < 0.001). Using ACTH concentrations in January as the comparative time point, there were significant differences in ACTH concentrations when January was compared to all months except December (*P* = 0.56). In July, median plasma ACTH increased to 52.9 (29.7–214.0) pg/ml and continued to increase on a monthly basis until reaching a maximum median concentration in September [median 122.0 (41.7–319.0) pg/ml]. Plasma ACTH concentrations declined in November to 32.1 (12.4–91.3) pg/ml and returned to low concentrations of 13.2 (5.3–73.6) pg/ml in December. Median ACTH concentrations were further organized into the months grouped together by the Equine Endocrinology Group for diagnosis of PPID from baseline ACTH concentrations for comparison to current horse guidelines (Table 1) (3).

**Table 1:** ACTH concentrations in the current study and other studies in donkeys compared to the 2021 Equine Endocrinology Group (EEG) recommendations for seasonal interpretation of baseline ACTH.

	**ACTH concentrations from donkeys unlikely to have PPID**			**EEG cut-offs for PPID diagnosis in horses** ^**a, b**^		
Seasonal interpretation of results, baseline ACTH (pg/ml)	Current study^b^	Gehlen et al. (18)^c^	Dugat et al. (8)^d^	PPID unlikely	Equivocal	PPID likely
	Median (range)	Median (range)	Mean ± *SD*			
December–June	17.3 (5.0–80.5)	Feb, May, Nov	May, June	<15	15–40	> 40
		15.1 (5–72.6)	66.7 ± 20.7			
July and November	40.9 (12.4–214.0)			<15	15–50	>50
August	88.1 (46.0–259.0)	49.55 (19.5–143)		<20	20–75	>75
September–October	97.0 (35.7–319.0)			<30	30–90	>90

^a^*https://sites.tufts.edu/equineendogroup/files/2021/12/2021-PPID-Recommendations-V11-wo-insert.pdf*.

^b^*Chemiluminescence immunoassay, Immulite 1000*.

^c^*Chemiluminescence immunoassay, Immulite 2000 XPi*.

^d^*Assay details not available*.

With regards to months with two sampling time points, ACTH concentrations from the middle of June (first sampling point) were significantly higher than concentrations from the end of June (second sampling point), with a median difference of 15.3 pg/ml [median concentration of 19.8 (12.5–56.4) vs. 33.9 (21.8–80.5), respectively; *P* < 0.001]. There were no significant differences in ACTH concentrations in the middle of the month compared to the end for the months of July, August, and September. Concentrations from the middle of the month in October and November were significantly higher than at the end of the month, with a median difference of 28.9 pg/ml in October and 10.5 pg/ml in November (*P* < 0.001).

### Interaction effects

**Figure 2:**
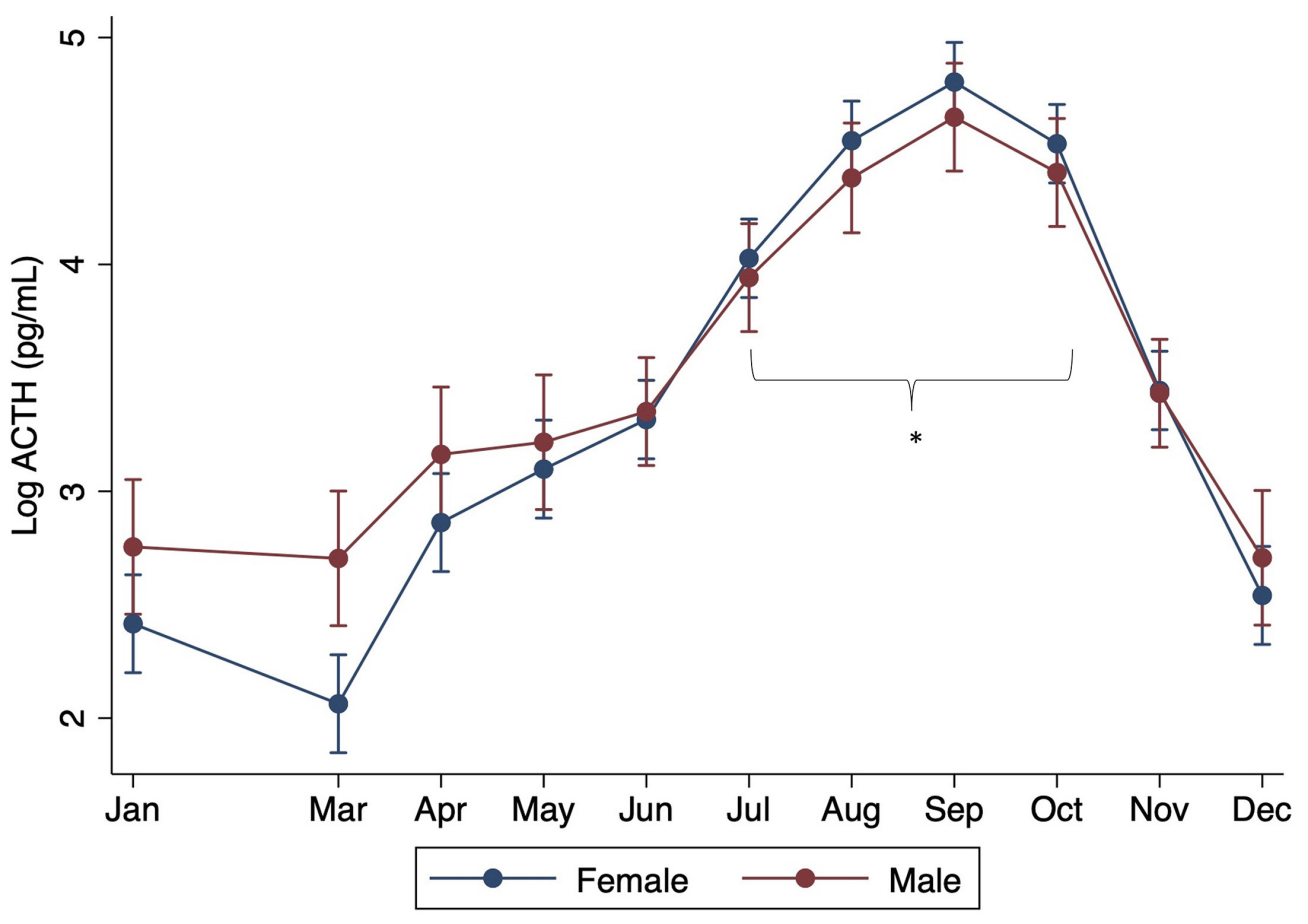
Effect of sex on plasma ACTH concentrations by month. Log of the median (range) shown. Asterisks denote months in which there was a statistically significant difference between gelded male and intact female donkeys.

**Figure 3:**
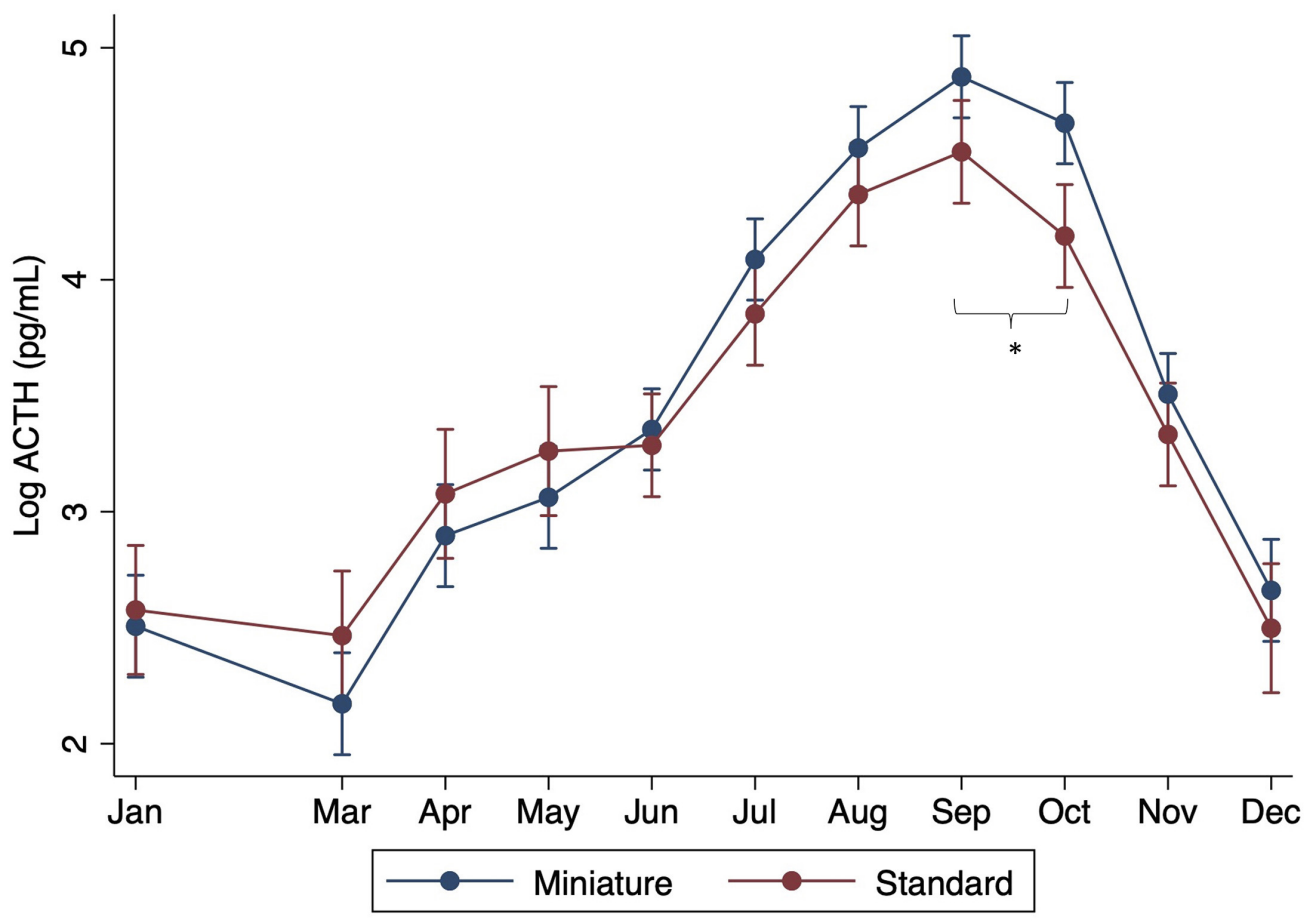
Effect of donkey size (standard vs. miniature) on plasma ACTH concentrations by month. Log of the median (range) shown. Asterisks denote months in which there was a statistically significant difference between standard and miniature donkeys.

When controlling for month, there were no significant differences found with breed, age, or sex (*P* > 0.05). However, significant interactions were found between sex and month, with males having significantly lower ACTH concentrations in July through October compared to females (*P* < 0.001) (Figure 2). Significant interactions were also found between month and size, with standard donkeys having lower ACTH concentrations in September and October compared to miniatures (*P* < 0.001) (Figure 3). Finally, a significant interaction was observed between month and age (*P* < 0.001), with older donkeys having higher ACTH concentrations in August and September.

### Complete blood counts

Baseline CBCs in March were largely within normal limits. Two standard donkeys had elevated eosinophil counts. The highest eosinophil count (3,246 cells/μ*l*) was associated with the ACTH concentration near the high end of the reference range for the month of March (38.1 pg/ml; median 9.2 pg/ml). The other donkey with a mildly increased count (1,081 cells/μ*l*) had an ACTH concentration of 6.8 pg/ml. Two donkeys had very mildly increased neutrophil counts ranging from 6,553 to 7,259 cells/μ*l*, considered clinically insignificant and associated with ACTH concentrations of 18.9 and 4.0 pg/ml, respectively. Repeat CBCs in September showed persistent eosinophilia in the two donkeys from March, with an improved count of 944 cells/μ*l* associated with an ACTH of 148 pg/ml and 1394 cells/μ*l* associated with an ACTH of 122 pg/ml (median for that time point of 119 pg/ml, upper range 295 pg/ml). One additional standard donkey had a minimal lymphopenia, and a miniature donkey had a minimal neutropenia, with ACTH concentrations of 128 pg/ml and 93 pg/ml, respectively.

## Discussion

This study showed that adult donkeys have seasonal increases in plasma ACTH concentrations, similar to the circannual variation reported in horses. Plasma concentrations began increasing in July, peaked in September, and remained elevated while trending downward through November. Additionally, donkey plasma ACTH concentrations were higher than those reported for horses in the summer and fall, most notably the months of July through November (1). This supports the hypothesis that donkeys exhibit a larger seasonal increase in ACTH relative to horses. These findings complement the previous studies assessing ACTH concentrations in donkeys, with the frequent, serial sampling in this study providing a more thorough evaluation of the seasonal changes in donkeys and providing a necessary step toward establishing reference ranges in this species (8, 22).

According to the most recently published guidelines on PPID in horses by the Equine Endocrinology Group, our population of donkeys would more often be classified as either equivocal or likely to have PPID if using their baseline plasma ACTH concentrations alone (Table 1) (1). ACTH concentrations in the donkeys remained within the cut-off established for unlikely diagnosis of PPID in horses for the months of December, January, March, and April. The donkeys' median plasma ACTH concentrations fell within the equivocal range in May and June (equivocal range 15–40 pg/ml for those months) as well as November (15–50 pg/ml). The donkeys' plasma ACTH concentrations rose above equivocal and into the range of likely PPID in July, August, September, and October. Based on these guidelines and our data it would be reasonable to measure plasma ACTH concentrations in donkeys and use the Equine Endocrinology Group's guidelines in winter through mid-spring (April). However, it is important to note that the assay referenced in the 2021 Equine Endocrinology Group is the Immulite 2000 XPi, which has lower ACTH concentrations than other analyzers, including the Immulite 1000; other assays may have different bias and require assay-specific cut-off concentrations (1).

Additionally, animals should be tested only if there is a clinical suspicion of an endocrinopathy, and not in clinically healthy animals as in this research. One challenge with the current study is that, while the donkeys appeared clinically healthy and of an unlikely age to have PPID, a subset could have had early PPID or other factors contributing to increased ACTH concentrations. A recent study found that dynamic testing utilizing TRH stimulation as a more sensitive tool for diagnosing PPID is useful in donkeys; however, further research is required to determine reference ranges and TRH testing was not performed in this herd (23).

Amongst our population of donkeys there was also a wide range of plasma ACTH concentrations in the summer and fall, with a few individuals approaching single, sporadic concentrations of up to 300 pg/ml. Our donkeys were individually chosen to avoid phenotypes suggestive of PPID (based on age and physical examination). No single donkey in the study population displayed consistently high ACTH concentrations, and the donkeys with the highest ACTH concentrations were younger animals (ranging from 3 to 6 years old). Other factors that can elevate ACTH concentrations apart from PPID include stress and systemic illness. A study comparing donkey ACTH and cortisol concentrations with horse ACTH and cortisol concentrations before and after trailering found that donkeys exhibited an increase in both ACTH and cortisol concentrations, while horses only increased their cortisol concentrations (24). It is unknown whether venipuncture may cause a similar rise in ACTH concentrations. The donkeys displayed variable levels of stress at handling, although the individuals with the most resistance to venipuncture were not consistently among those with the highest plasma ACTH concentrations. The same individual researchers and handlers performed the venipunctures throughout the study to limit variation in handling practices. However, stress cannot be ruled out as a contributing factor to the wide range in plasma ACTH elevations, particularly in summer and fall months when ACTH concentrations were naturally on the rise. All individuals were deemed apparently healthy based on physical examinations performed at 6-month intervals and showed no significant change in their status throughout the study, so systemic illness was an unlikely contributor. However, additional diagnostics were not performed to verify their apparent health such as a biochemistry panel, baseline or dynamic insulin testing, fecal flotation for parasites or urinalysis.

Other limitations of this study included the small population size and greater proportion of females. The statistically significant differences in castrated males vs. females in July through October, as well as the differences between standard and miniature donkeys in September and October, require additional studies to investigate further. With regards to size, depending upon the study, pony breeds have been shown to be at similar risk or more likely to develop PPID compared to horses (3). It has been hypothesized that particular pony breeds may be at greater risk, and the same may hold true for miniature donkeys (3). Ideally a larger population of both miniature and standard donkeys, with a more balanced group of males and females, would be performed. However, confounding factors that could influence ACTH concentrations were minimized by sampling donkeys kept at the same property under the same management practices and were consistently sampled at the same time of day.

An additional limitation was the possibility of batch effect, as samples were shipped and analyzed on a monthly basis, as well as the change in assay part way through the study period. The Immulite 1000, used for samples in March through May, yields concentrations higher than those provided by the Immulite 2000 XPi, and a mathematical calculation was used to convert concentrations derived from the Immulite 1000 so they were able to be appropriately compared with concentrations from the rest of the year. Furthermore, it is known that geographic latitude and longitude and time of day affect ACTH concentrations in horses (4, 25). A similar phenomenon is possible in donkeys as well. A recent study performed assessing median ACTH concentrations in donkeys in Texas in May and June had values roughly three times higher than the median concentrations found in this study in California in those same months (8). This highlights the marked variation that can occur, whether by location, age, husbandry, assay used, or other factors, and the need for further study with more animals, as well as geographic-specific references. In the current study, circadian fluctuations in ACTH concentrations on each sampling day were controlled for by sampling in the morning within the same window of time, however sampling time can also play a role in establishing reference ranges. Finally, ACTH concentrations were obtained bi-monthly for summer and fall months but once monthly for other months. The increased sampling was done to better track the rise in ACTH concentrations over summer and fall, however this also resulted in some incongruity in comparing concentrations across months. Collecting bi-monthly samples throughout the year would have been a preferred study design.

In conclusion, this study has clinical implications for management of donkeys. Based on the results, the authors suggest that donkeys could be screened for PPID with basal ACTH concentrations from December through April, using the most recent guidelines supplied by the Equine Endocrinology Group and their referenced assay, until suitable reference ranges for the rest of the year are established. This will avoid the potentially large and variable increase in plasma ACTH concentrations that our group of donkeys exhibited in July through November that will make interpretation difficult. The caveat to this recommendation is that early stages of PPID may be overlooked without dynamic testing, similar to horses. Ultimately, this study needs to be repeated in a larger population and across various latitudinal zones, to establish seasonal and latitudinal reference ranges for basal ACTH concentrations in donkeys.

## Data availability statement

The raw data supporting the conclusions of this article will be made available by the authors without undue reservation upon request.

## Ethics statement

The animal study was reviewed and approved by UC Davis IACUC. Written informed consent was obtained from the owners for the participation of their animals in this study.

## Author contributions

SH and EB designed the study, organized and conducted sample acquisition, interpreted and analyzed results, and wrote and revised the manuscript. PK statistically analyzed results and participated in manuscript writing and revision. KGM aided in organization of sample acquisition and participated in manuscript writing and revision. EG participated in acquisition and interpretation of results and participated in manuscript writing and revision. All authors contributed to the article and approved the submitted version.

## Funding

Funding was provided by the Center for Equine Health at UC Davis (grant 3-V435SES), the Animal Health Diagnostic Center Endocrinology Laboratory at Cornell University, and the Roberta A. and Carla Henry Endowed Chair in Emergency Medicine and Critical Care at UC Davis.

## Conflict of interest

The authors declare that the research was conducted in the absence of any commercial or financial relationships that could be construed as a potential conflict of interest.

## Publisher's note

All claims expressed in this article are solely those of the authors and do not necessarily represent those of their affiliated organizations, or those of the publisher, the editors and the reviewers. Any product that may be evaluated in this article, or claim that may be made by its manufacturer, is not guaranteed or endorsed by the publisher.

## Abbreviations

ACTH: adrenocorticotropic hormone
PPID: pituitary pars intermedia dysfunction.
